# WebCMap: an R package for high-throughput connectivity analysis within the CMap framework

**DOI:** 10.1093/bioadv/vbaf278

**Published:** 2025-11-05

**Authors:** Hongen Kang, Yin-Ying Wang, Peilin Jia

**Affiliations:** China National Center for Bioinformation, Beijing 100101, China; Beijing Institute of Genomics, Chinese Academy of Sciences, Beijing 100101, China; China National Center for Bioinformation, Beijing 100101, China; Beijing Institute of Genomics, Chinese Academy of Sciences, Beijing 100101, China; China National Center for Bioinformation, Beijing 100101, China; Beijing Institute of Genomics, Chinese Academy of Sciences, Beijing 100101, China

## Abstract

**Motivation:**

Experimentally generated drug-induced transcriptomic signatures are valuable resources to infer candidate drugs for unseen transcriptomes. The Connectivity Map (CMap) includes over 720 000 compound-induced signatures and has been widely used in drug repurposing. However, the computational resources required for an unbiased screen across all these signatures, along with the inconsistent results from different methods, presented huge challenges for the connectivity analyses.

**Results:**

In this study, we developed WebCMap, an R package to search for candidate compounds with similar or reverse activities across all CMap drug-induced signatures. WebCMap implements six widely used methods and a meta-score to evaluate the consistency among these methods. Through a web-accelerated framework, pre-calculated statistics for the permutation test, and multi-core parallelization, WebCMap enables fast screening and retrieval of the results on personal computers within a reasonable time.

**Availability and implementation:**

WebCMap is available at https://github.com/geneprophet/WebCMap.

## 1 Introduction

The Connectivity Map (CMap) is a comprehensive resource for drug-induced transcriptomic signatures (Lamb *et al.* 2006). In this context, a signature refers to the genome-wide differential expression pattern that characterizes the cellular response to a perturbation. Specifically, a signature is represented as a vector of gene-level *Z*-scores, derived from comparing perturbation and control conditions to quantify both the direction and magnitude of transcriptional changes. Depending on the study context, these signatures may be described as drug-induced signatures (e.g. drug versus dimethyl sulfoxide control), disease signatures (e.g. case versus non-disease control), or other context-specific perturbational profiles. CMap and the Library of Integrated Network-Based Cellular Signatures (LINCS) (Subramanian *et al.* 2017) currently contain over 720 000 drug-induced signatures derived from perturbations using >30 000 compounds across >70 cell lines. The breadth and coverage of drugs, compounds, and the tested cell lines made CMap and LINCS valuable resources for drug–drug, compound–compound, and disease–drug studies (Pushpakom *et al.* 2019, Zhang *et al.* 2021, Meier *et al.* 2025). However, such a vast volume of signatures also poses computational challenges for connectivity analyses.

Following the convention of CMap, we use the connectivity analysis to refer to methods that evaluate the correlations between gene expression signatures of different perturbagens. Typically, a pair of signatures to be compared includes one from experimental perturbation with known compounds (e.g. reference signatures from CMap) and the other from an unknown perturbation (e.g. query). Positive correlations indicate there are likely shared mechanisms of action (MoA), whereas negative correlations imply that the compound of the reference signature can be used to reverse the query signature, and hence, can be considered as candidate drugs to treat the latter. So far, several methods have been published for the connectivity analysis (Samart *et al.* 2021). Depending on the genes used, we categorized these methods into two major groups: one using all measured genes of the query signature, while the other uses only the extreme genes, e.g. those ranked at the top or bottom parts of the whole gene list. For example, the official CMap platform utilizes a strategy based on Gene Set Enrichment Analysis (GSEA) for the connectivity analyses, i.e. comparing the extreme genes of the query signature against the whole gene list of a CMap signature. This method is computationally intensive for hundreds of thousands of signatures and thus, it only allows analyses for 2837 signatures in the touchstone dataset derived from nine core cell lines. Alternatively, Signature Commons (SigCom) LINCS proposes a fast web search engine by performing the Mann-Whitney U test (Evangelista *et al.* 2022) to assess if the input gene set is primarily located at the top or bottom of the ranks in a drug-induced signature. Moreover, iLINCS applies extreme Pearson’s correlation of signed gene-level significance to connect query and reference signatures (Pilarczyk *et al.* 2022). The R package *signatureSearch* supports both enrichment analysis (Fisher’s exact test) and correlation-based connectivity analysis (Duan *et al.* 2020). More recently, Marino *et al.* introduced the LINCS L1000 Signature Search (L2S2) web server using Fisher’s exact test to identify compounds or gene perturbations that produce concordant or discordant expression profiles (Marino *et al.* 2025). Several benchmark studies have compared these methods (Cheng *et al.* 2014, Lin *et al.* 2020, Samart *et al.* 2021, Struckmann *et al.* 2021). Overall, the performance of these methods is context-dependent, with no single method consistently outperforming all others in different scenarios.

**Figure 1.**
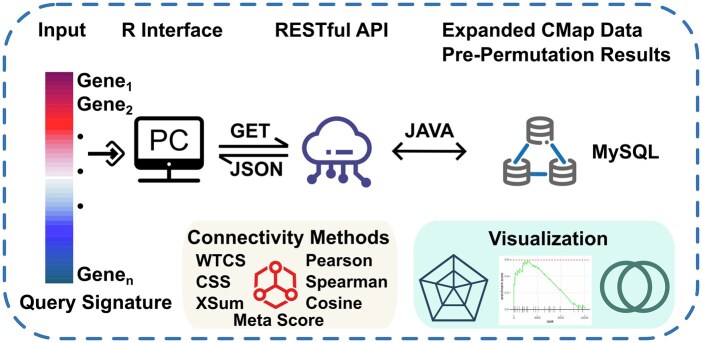
Schematic overview of WebCMap. Diagram showing the WebCMap R package interfacing with a remote cloud-based API connected to a database storing expanded CMap signatures and pre-permutation results. The workflow includes six connectivity analysis methods and a meta-score for consensus evaluation, with results visualized by radar, Venn, and GSEA plots. CMap, connectivity map; PC, personal computer; RESTful, representational state transfer; API, application programming interface; WTCS, weighted connectivity score; CSS, connection strength score; XSum, the eXtreme Sum score.

Here, we present WebCMap, an R package for accelerated connectivity analyses through a web-optimized computational framework to allow assessment of the whole set of the CMap signature repository (Fig. 1). WebCMap implements six connectivity methods: three using the extreme genes of the query signature (i.e. Weighted Connectivity Score or WTCS, Connection Strength Score or CSS, and eXtreme Sum or XSum) and three using the whole gene list (i.e. Spearman correlation, Pearson correlation, and Cosine similarity). It also introduces a meta-score that combines the significance derived from all six methods to assess the consistency among methods (Kang *et al.* 2024). We enhance computational efficiency using three key strategies. First, WebCMap dynamically retrieves reference signatures from cloud-hosted Representational State Transfer (RESTful) Application Programming Interface (API) during the analyses to effectively decouple memory-intensive operations from local computation. Second, for the computation of non-parametric *P*-values for CSS and XSum, we pre-permuted each reference signature 10 000 times and calculated the statistics for CSS and XSum for each iteration to construct the background matrix. This matrix was then stored in a MySQL database hosted by our WebCMap server, which greatly reduced the computation time for each query. Third, we used multi-core parallelization to optimize the computation processes. In sum, WebCMap allows connectivity analyses with the full set of signatures, offering high efficiency and low computational resource requirements.

## 2 Implementation

### 2.1 Connectivity methods

We downloaded the expanded CMap data (level 5), including 720 216 signatures for 12 327 genes. These formed the reference signature repository for WebCMap. For any query transcriptome, we implement six connectivity methods to identify the reference signatures that showed positive or negative associations. Details of these methods are presented in Table 1, available as supplementary data at *Bioinformatics Advances* online and also in our previous work (Kang *et al.* 2024). Among them, WTCS, CSS, and XSum use a hyperparameter K to define extreme genes for the calculation, often set as 50. In practice, users can select K as 100, 150, or 200. In addition, the calculation of SCC, PCC, and Cosine similarity is quite fast, returning results within one second for a query transcriptome. However, CSS and XSum use a permutation test to calculate the empirical *P*-values and take much longer to generate results, e.g. ∼100 h are required for a query transcriptome to be compared with all the signatures in CMap. To reduce the computational time, we generated 10 000 random sets for each of the 720 216 signatures in CMap and calculated the corresponding CSS and XSum values for each random set ahead of time. These random sets are used to calculate the nonparametric *P*-values by CSS and XSum, where the observed CSS and XSum values of the query signature are compared against the precomputed statistics generated from the random set. This operation reduces the computation time for CSS and XSum to within one second per signature pair. Finally, the WTCS calculation still requires ∼5 s per query-reference pair. Thus, we restricted the WTCS analyses to only those that have been determined as significant by any of the other five methods to save computational time. Finally, we propose a meta-score to assess the consistency of results across all six methods. The meta-score is calculated by counting how many of the methods yield results within the top 5% of all outcomes, with each method contributing a vote (0 or 1). Consequently, the meta-score ranges between 0 and 6, where 0 indicates that none of the methods produce a top 5% result, and 6 indicates that all six methods rank the query-reference signature pair as within the top 5% for the corresponding results. The overall processing steps were illustrated in Fig. 1, available as supplementary data at *Bioinformatics Advances* online. Notably, WebCMap leverages parallel processing on multi-core processors to perform connectivity analysis efficiently. Finally, we provide three plotting functions for visualization of the results, including the radar, GSEA, and Venn plots.

### 2.2 Web-based RESTful API

We allocated a local MySQL database and a web server to support the data and analyses by WebCMap. Reference signatures were formatted as JSON files and indexed to improve the search engine’s performance. The system architecture was implemented utilizing Java and the Spring Boot framework. Spring facilitated seamless database connectivity with MySQL and enabled the connection of RESTful APIs to handle concurrent user requests, allowing >100 000 simultaneous connections.

We provide two functions for the connectivity analyses: *run_negative_CMap* and *run_positive_CMap*. The former identifies candidate reference signatures showing negative relationships with the query signature. Thus, this function is mainly used for drug repurposing. The computational time is about 40 min with a variation of <5 min using personal computers (PCs) physically located in China. The computational time ranges from 100 to 120 min for PCs located in the United States, likely due to the network transmission distance between the local PCs and our WebCMap server.

The *run_positive_CMap* function screens for compounds in the reference signatures showing similar gene expression patterns to the query signature. Such positive relationships are often used to infer mechanisms of action or potential side effects of novel drugs.

## 3 Example application

For the negative relationship, we used the genome-wide association study (GWAS) data for bipolar disorder (Mullins *et al.* 2021) as an example to demonstrate how WebCMap can be used to infer candidate compounds. The rationale was that, based on the GWAS results, we could impute the genetically regulated expression (GReX) using the transcriptome-wide association study (TWAS) method for the disease. Specifically, we utilized the S-PrediXcan algorithm in conjunction with the multivariate adaptive shrinkage model to generate GReX for bipolar disorder (Barbeira *et al.* 2018, Urbut *et al.* 2019). The results were presented as a ranked list of genes by their TWAS *Z*-scores and used as input to WebCMap. The example input was also available via the function “*data(query_signature)*” in WebCMap and as Table 2, available as supplementary data at *Bioinformatics Advances* online. Then, we applied the *run_negative_CMap* function with the default parameters. This application yielded 601 candidate signatures (Table 3, available as supplementary data at *Bioinformatics Advances* online), each annotated with the canonical simplified molecular input line entry system (SMILES), cell line, dosage, exposure time, and other experimental conditions for better interpretability of the results. In this case, SC-12267 (vidofludimus) was ranked the highest among all candidate compounds, with a meta-score of 5. Previous work (Kim *et al.* 2025) had investigated SC-12267 in clinical trials for multiple sclerosis, a chronic demyelinating disorder of the central nervous system. Considering the potential relationship between multiple sclerosis and psychiatric comorbidities (Joseph *et al.* 2021), SC-12267 may represent a promising therapeutic candidate for bipolar disorder. The results were also visualized through a radar plot (Fig. 2, available as supplementary data at *Bioinformatics Advances* online), a GSEA plot (Fig. 3, available as supplementary data at *Bioinformatics Advances* online), and a Venn plot (Fig. 4, available as supplementary data at *Bioinformatics Advances* online).

To evaluate the method for positive connectivity analysis, we selected the RICTOR gene knockdown signature as an example (Table 4, available as supplementary data at *Bioinformatics Advances* online). RICTOR is a core component of the mTOR signaling pathway. Among all CMap signatures, there were 9608 signatures generated by 64 mTOR inhibitors. Thus, the RICTOR-knockdown signature was expected to show positive similarities with the mTOR inhibitor signatures. Applying the *run_positive_CMap* function, we identified 82 966 significant signatures involving 12 727 compounds (Table 5, available as supplementary data at *Bioinformatics Advances* online), among which 34 compounds were from the 64 mTOR inhibitors. Using Fisher’s exact test, we confirmed that the mTOR compounds were significantly enriched in the candidate signatures (*P* = 2.5 × 10^−9^). Importantly, four compounds had a meta score of 6, implying that the strategy for positive relationships was effective. The top-ranked compounds represent the most robust candidates, although interpretation should consider context-specific factors such as cell type, dosage, and exposure time.

## 4 Conclusion and discussion

We presented an R package, WebCMap, to greatly accelerate connectivity mapping for drug repurposing. It reduces the heavy reliance on high-performance computing resources and decreases the computational time by leveraging several strategies, including pre-calculated permutation data and multi-core parallelization. This enables analyses using the full list of CMap signatures. Although WebCMap was designed to be lightweight and compatible with PCs, its framework can be readily scaled to high-performance computing servers or cloud environments. In such settings, computational efficiency can be further enhanced by exploiting more CPU threads in parallel and taking advantage of wider network bandwidth, thereby substantially reducing the runtime for large-scale batch analyses. We anticipate that WebCMap will be extensively used by researchers across a wide range of fields.

## Supplementary Material

vbaf278_Supplementary_Data

## Data Availability

The data and code underlying this article are available on *GitHub* at https://github.com/geneprophet/WebCMap.
