# Supplementary material for: WebCMap: an R package for high-throughput connectivity analysis within the CMap framework
Source: Bioinform Adv. 2025 Nov 5;5(1):vbaf278. doi: 10.1093/bioadv/vbaf278 (PMC12629228; doi:10.1093/bioadv/vbaf278)
Supplement: vbaf278_Supplementary_Data [file vbaf278_supplementary_data.zip › WebCMap_Supplement.docx]

**Table S1. Summary of the methods for the connectivity analysis implemented by WebCMap**

| **Full Name** | **Abbreviation** | **Group** | **Definition** |
| --- | --- | --- | --- |
| Weighted Connectivity Score  (PMID: 29195078) | WTCS | Extreme-gene approach | WTCS is a non-parametric method based on the weighted Kolmogorov-Smirnov enrichment statistic (ES). |
| Connection Strength Score  (PMID: 18518950) | CSS | Extreme-gene approach | CSS defines a connection strength score by comparing the ordered gene list in the reference signature and the K upregulated or downregulated genes in the query data. The significance of the connection score is assessed by 10,000 permutation data. |
| eXtreme Sum Score  (PMID: 25606058) | XSum | Extreme-gene approach | XSum defines a gene list for the reference compound signature including top K up-regulated and down-regulated genes (K can be 50, 100, 150 or 200). It also defines two gene lists for the query signature. Then, XSum sums up the compound gene expression values in the compound signature for the query genes. XSum uses 10,000 permutations to calculate a non-parametric p-value. |
| Spearman Correlation Coefficient  (PMID: 34013329) | SCC | Full-gene approach | The SCC metric quantifies the rank-based correlation between the reference profile and the query profile across the common set of genes between the two profiles. |
| Pearson Correlation Coefficient  (PMID: 34013329) | PCC | Full-gene approach | The PCC metric quantifies the linear correlation between the reference profile and the query profile across the common set of genes between the two profiles. |
| Cosine Similarity  (PMID: 34013329) | CS | Full-gene approach | The Cosine metric quantifies the cosine similarity between the reference profile and the query profile across the common set of genes between the two profiles. |


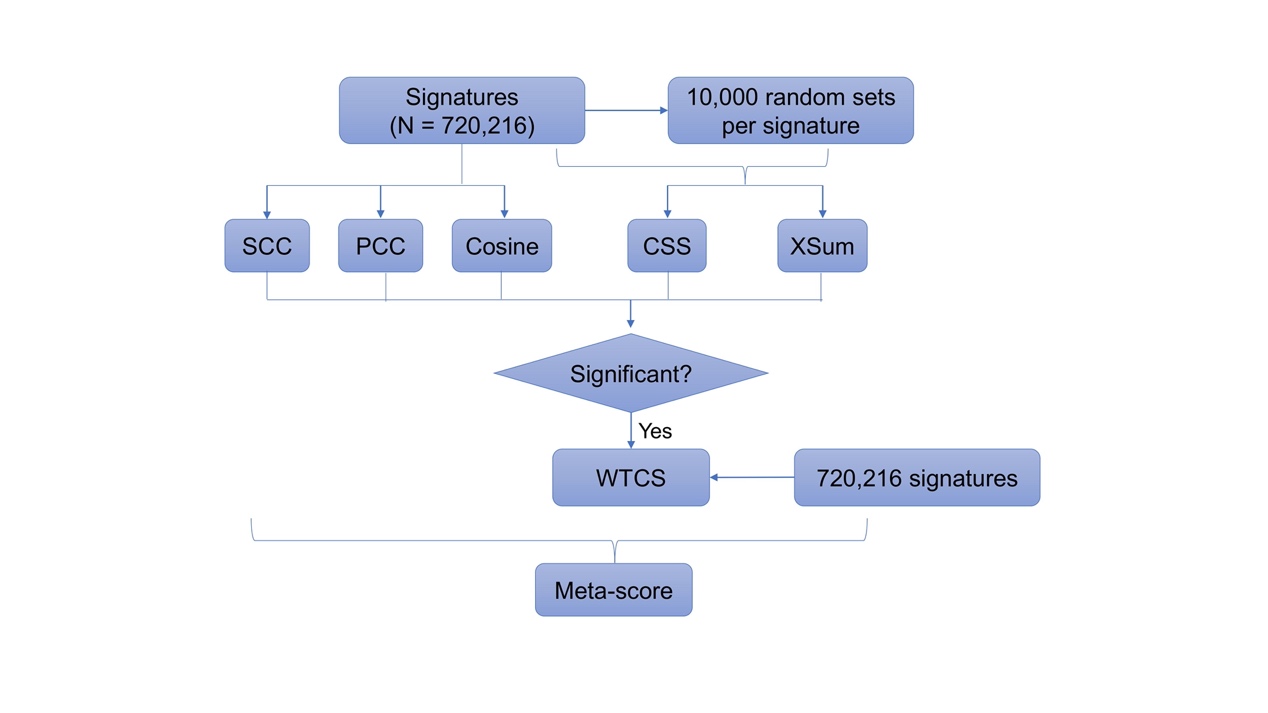


**Supplementary Figure S1.** The workflow diagram of the connectivity analysis in WebCMap.


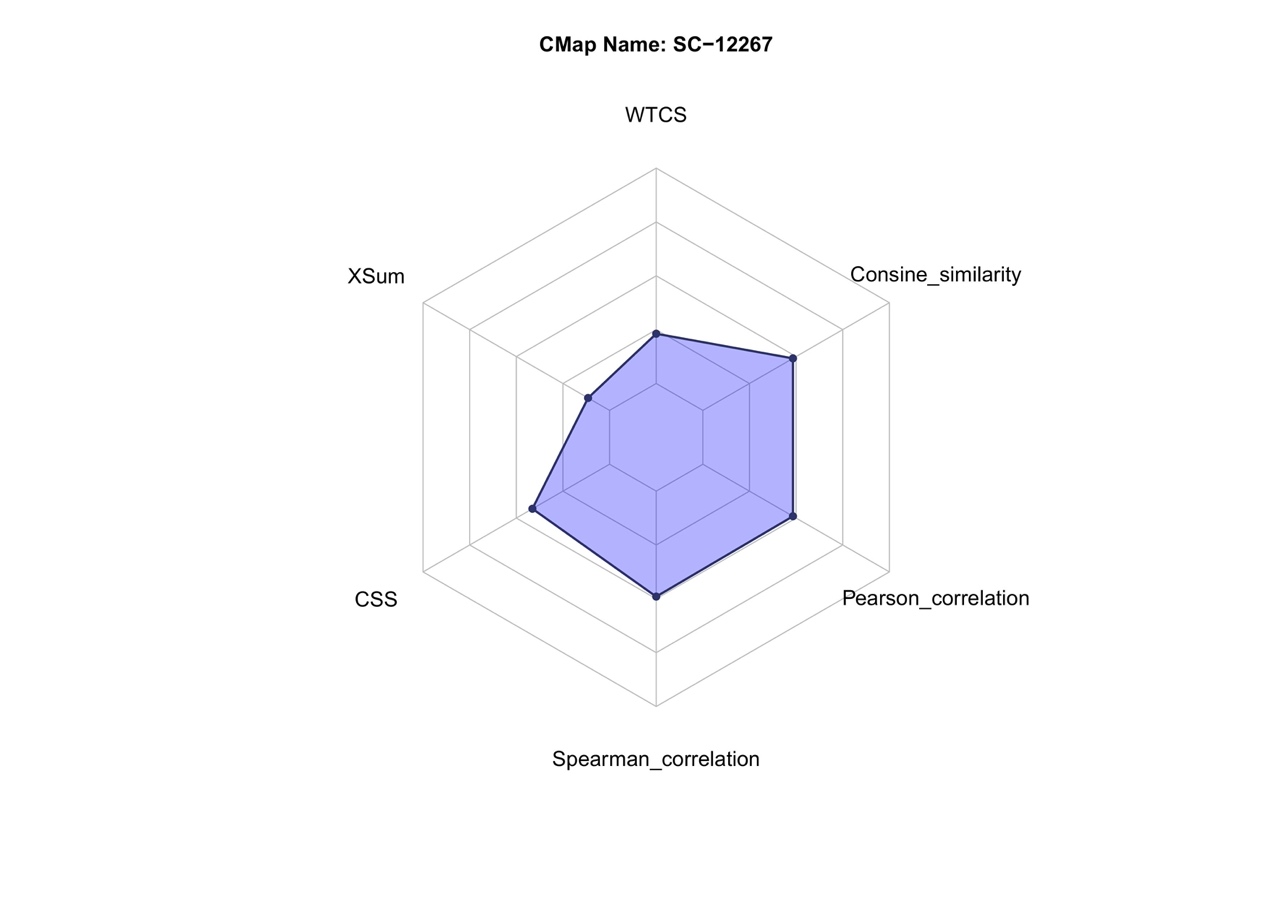


**Supplementary Figure S2.** An example radar plot using the compound SC-12267 for bipolar disorder.


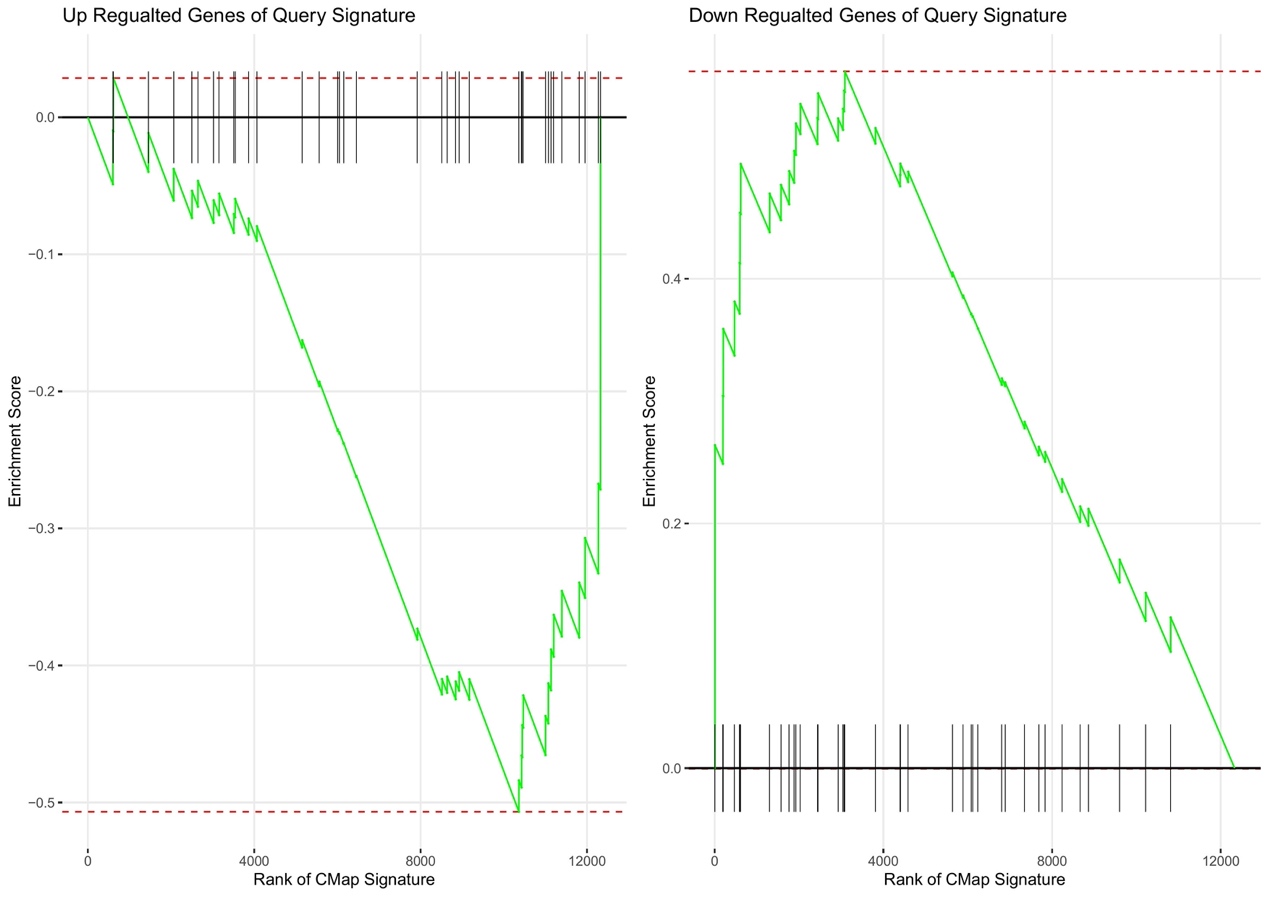


**Supplementary Figure S3.** An example GSEA (gene set enrichment analysis) plot.


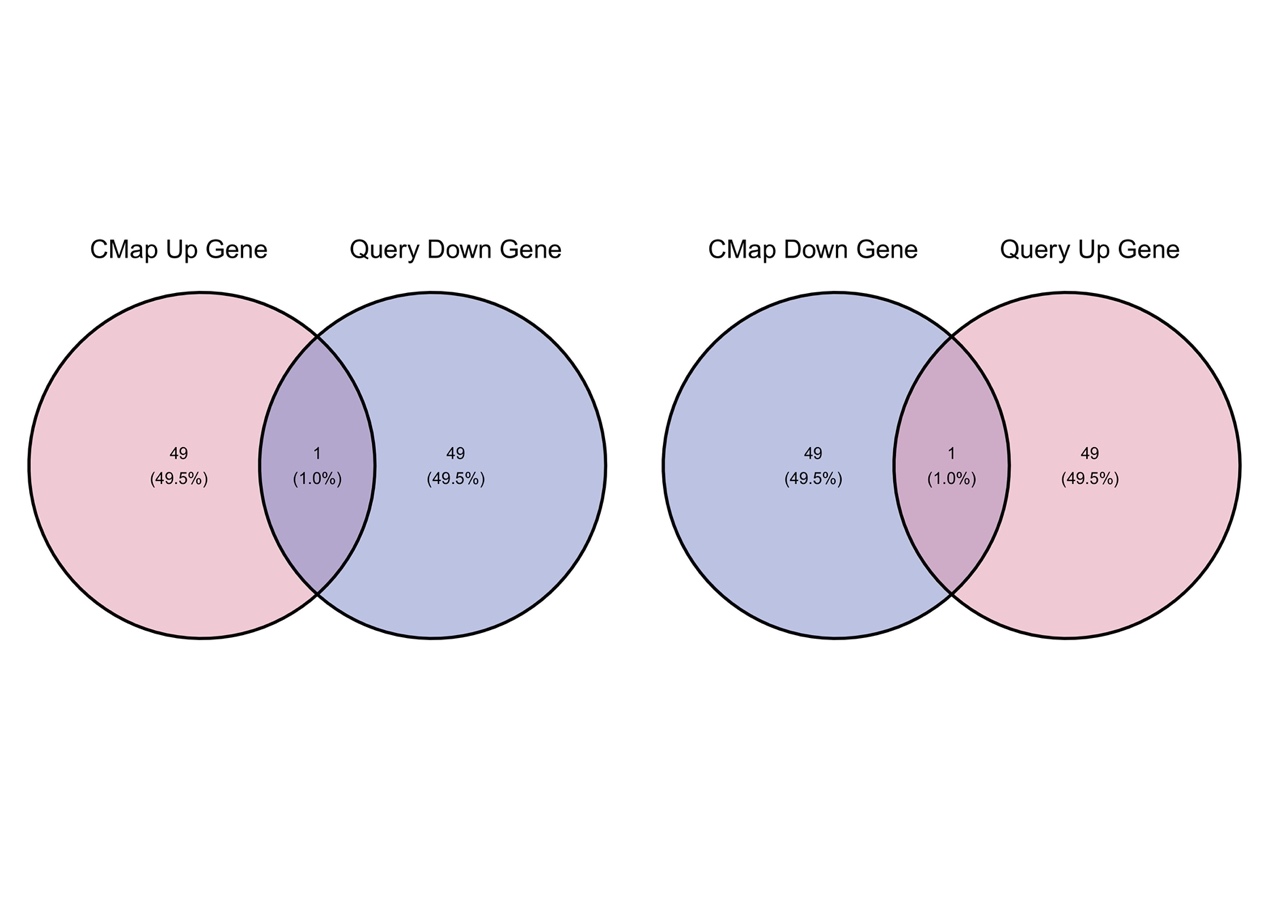


**Supplementary Figure S4.** An example Venn plot.
